# Supplementary material for: Plasticity-induced repression of Irf6 underlies acquired resistance to cancer immunotherapy in pancreatic ductal adenocarcinoma
Source: Nat Commun. 2024 Feb 20;15:1532. doi: 10.1038/s41467-024-46048-7 (PMC10879147; doi:10.1038/s41467-024-46048-7)
Supplement: Supplementary file 1 — Supplementary Information [file 41467_2024_46048_MOESM1_ESM.pdf]

Supplementary Information

Plasticity-induced repression of Irf6 underlies acquired resistance to cancer immunotherapy in pancreatic ductal adenocarcinoma

Il-Kyu Kim, Mark S. Diamond, Salina Yuan, Samantha B. Kemp, Benjamin M. Kahn, Qinglan Li, Jeffrey H. Lin, Jinyang Li, Robert J. Norgard, Stacy K. Thomas, Maria Merolle, Takeshi Katsuda, John W. Tobias, Timour Baslan, Katerina Politi, Robert H. Vonderheide, and Ben Z. Stanger

Supplementary Figures 1-13

Supplementary Tables 1-2

Supplementary Fig.1

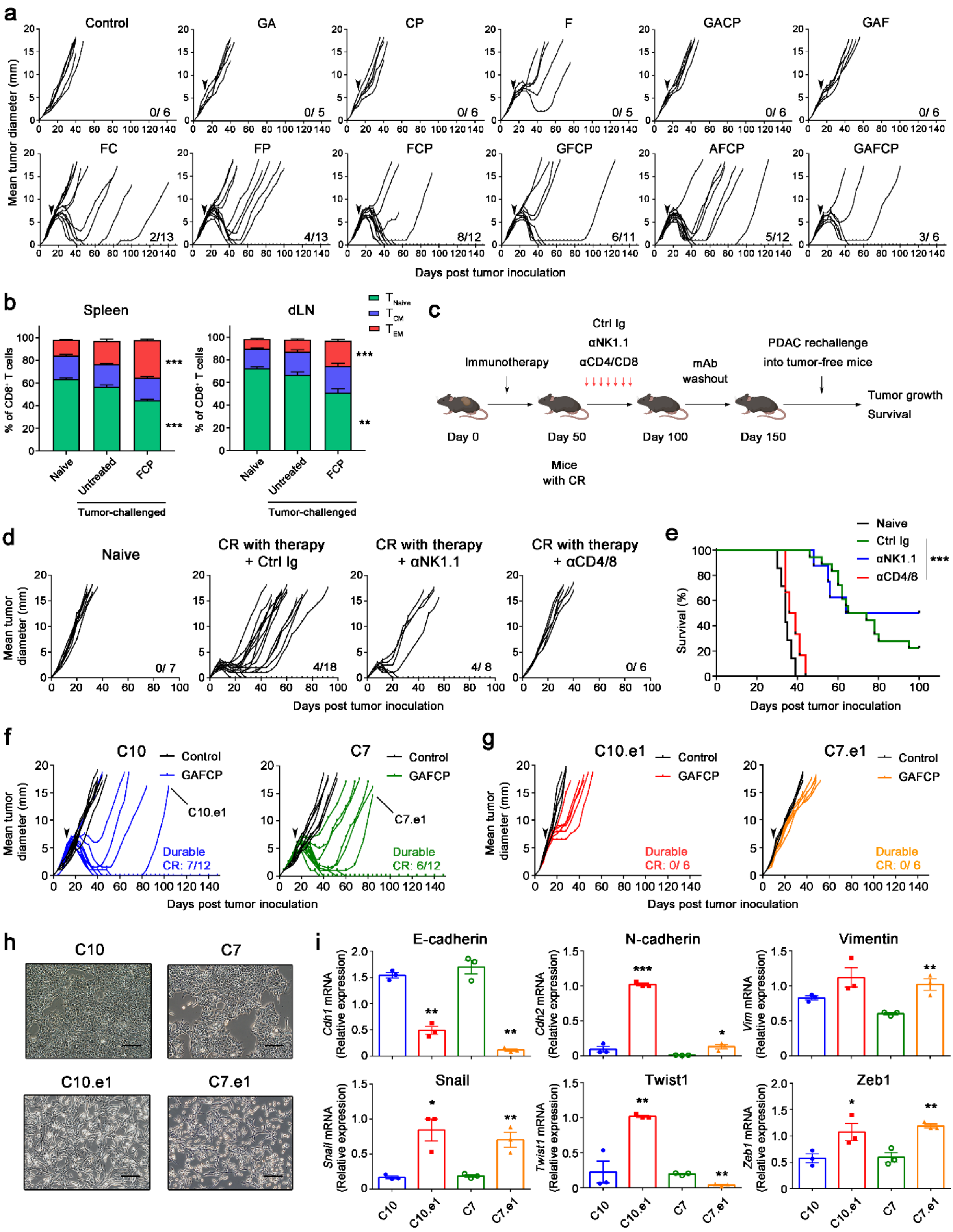

Supplementary Fig. 1 Tumor cell plasticity is a mechanism of acquired resistance to combination therapy in PDAC. a, Mice with established s.c. 4662 PDAC were treated with control IgG or indicated combinations of gemcitabine (G), nab-paclitaxel (A), αCD40 agonistic Ab (F), checkpoint blockades αCTLA-4 (C) and αPD-1 (P) Abs (arrow), and tumor growth was monitored (n = 5 to 13). Numbers in bottom right of tumor growth plots mean the number of mice with durable CR out of total mice in the group. **b**, The frequencies of T_{Naive}, central memory (T_{CM}), and effector memory T cells (T_{EM}) among CD8 T cells in the spleen (left) and draining lymph nodes (right) of naïve, tumor-challenged untreated and immunotherapy (FCP)-treated mice with CR (day 60) analyzed by flow cytometry (n = 3 for naïve, 6 for untreated, and 7 for FCP). **c**, A schematic illustration for assessment of T cell memory following therapy, created with BioRender.com. **d**,**e**, Mice with CR or near CR after therapy were treated with control IgG, αNK1.1 Ab, or αCD4 and αCD8 Abs through day 50 to 100. Following a washout period, mice were rechallenged with 4662 tumors on day 150 post initial tumor inoculation. Tumor growth (**d**) and survival (**e**) of mice treated as in **c** are shown (n = 6 to 18). Naïve mice challenged with an equivalent number of 4662 tumors are also shown as a control. **f**, Two independent tumor cell clones derived from 4662 PDAC by limiting dilution (C10 and C7) were injected s.c. into WT mice and treated with control IgG (n = 7 or 8) or GAFCP (arrow) (n = 12). Late Esc tumors that were harvested for cell line generation are noted (C10.e1 and C7.e1). **g**, Esc lines from s.c. implanted C10 and C7 tumors were transplanted again into WT mice, followed by control IgG (n = 3 or 4) or GAFCP (arrow) (n = 6). **h**, Representative bright field images of C10, C7, and Esc lines derived from these clones. Scale bars, 250 µm. **i**, Expression of EMT-related factors on C10, C7, and Esc lines derived from these clones were analyzed by qPCR (n = 3). Data were normalized to the amount of *Tbp* expression. * $P < 0.05$, ** $P < 0.01$, *** $P < 0.0001$ by Student's t test (**b**,**i**) and log-rank (Mantel-Cox) test (**e**). Source data and exact P value are provided as a Source Data file.

Supplementary Fig. 2

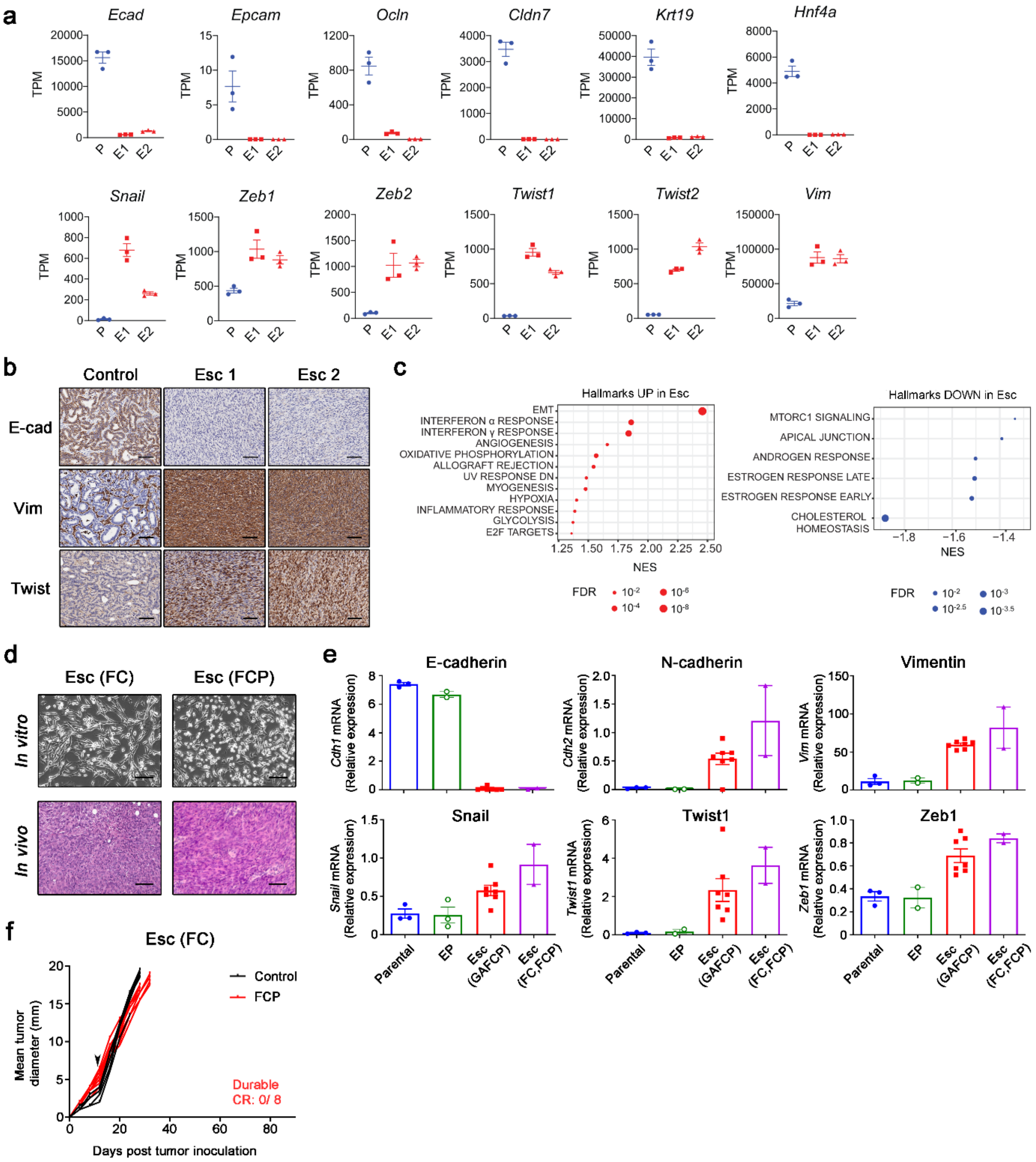

Supplementary Fig. 2 Acquired resistance to immunotherapy is associated with EMT. **a**, TPMs of key epithelial (top) and mesenchymal (bottom) genes in 4662 parental and two Esc cell lines. TPMs were calculated by Salmon. Each dot represents biological replicates. **b**, Representative IHC images of E-cadherin, Vimentin, and Twist in the original primary tumor tissues obtained from 4662 parental tumor-bearing mice with control IgG (Control) and the relapsed under combination therapy (Esc). **c**, GSEA of hallmark gene sets from the Molecular Signature Database summarized by FDR and NES. Hallmarks upregulated (left) and downregulated (right) in 4662 Esc vs. parental cell lines are shown. **d**, Representative bright field (top) and H&E (bottom) images of *in vitro* and *in vivo* Esc lines, generated under CD40 agonist plus anti-CTLA-4 (FC) or anti-CTLA-4 plus anti-PD-1 (FCP) treatment. Scale bars, 250 μm. **e**, Expression of EMT-related genes in 4662 parental (triplicate), EP, and Esc lines generated under chemoimmunotherapy (GAFCP) or immunotherapy alone (FC,FCP). Each datapoint in the EP and Esc plots represents averaged results from an individual cell line. **f**, Growth of 4662 Esc tumors generated under immunotherapy alone (FC), treated with control IgG or immunotherapy (FCP) (*n* = 5 for control and 8 for FCP). Source data are provided as a Source Data file.

Supplementary Fig. 3

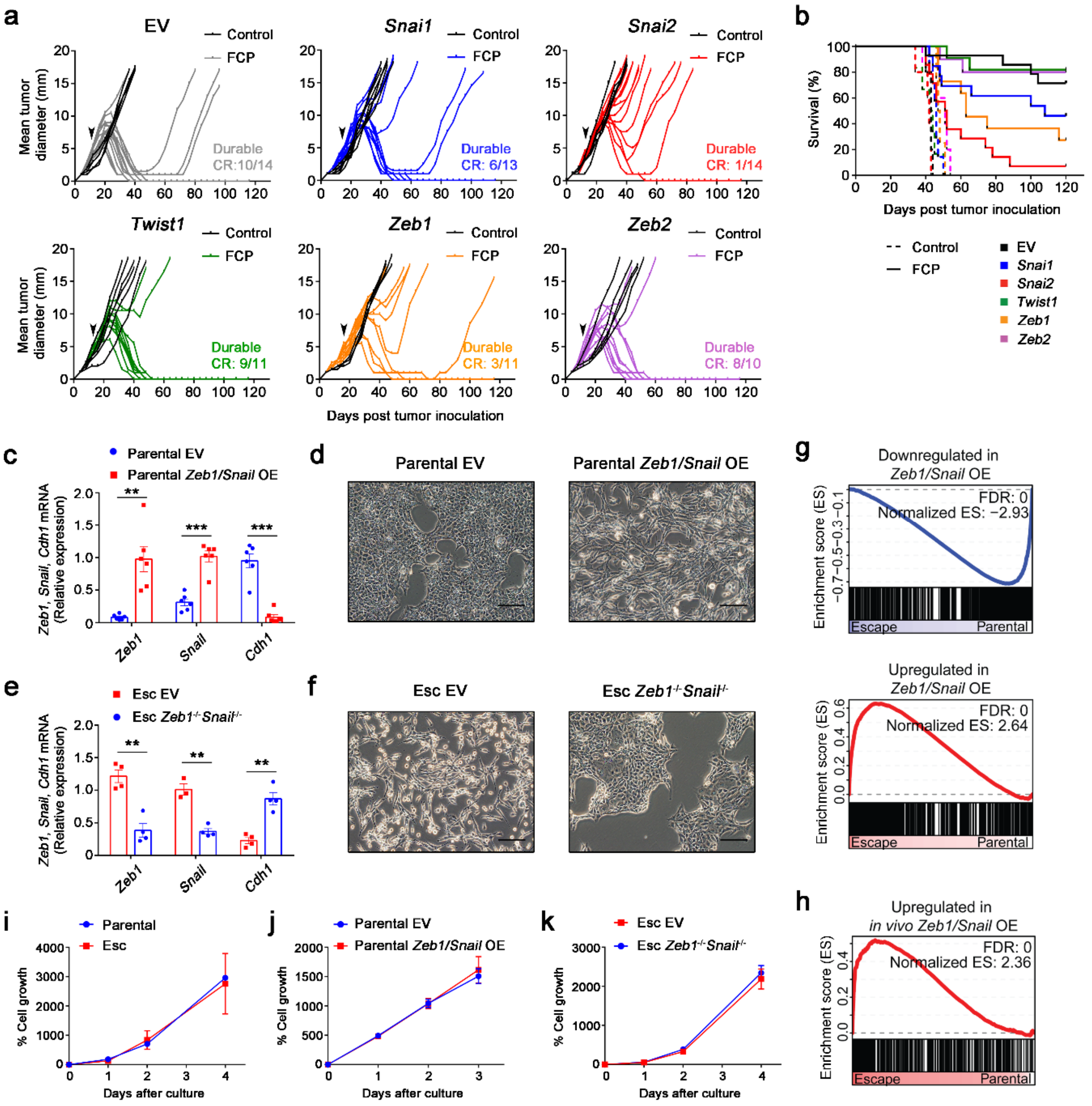

Supplementary Fig. 3 *Zeb1* and *Snail* drive resistance to immunotherapy. a,**b**, Tumor growth (**a**) and survival (**b**) of mice bearing clonal 4662 parental EV or each EMT-TF transduced tumors treated with control IgG or immunotherapy (FCP, arrow) (*n* = 10 to 14). **c**–**f**, Expression of *Zeb1*, *Snail*, and *Cdh1* (encoding E-cadherin) (**c**,**e**) and representative bright field images (**d**,**f**) of 4662 parental EV vs. *Zeb1*/*Snail* OE tumors and 4662 Esc EV vs. $Zeb1^{-/-}$ $Snail^{-/-}$ tumors are shown (*n* = 3 to 6). Scale bars, 250 µm. **g**, GSEA plots of gene signatures derived from 4662 parental cells overexpressing *Zeb1* and *Snail* (*Zeb1*/*Snail* OE) in 4662 parental vs. Esc lines. Gene signatures downregulated (top) and upregulated (bottom) with *Zeb1*/*Snail* OE are shown. **h**, GSEA plot of a gene signature derived from s.c. implanted *Zeb1*/*Snail* OE tumors in s.c. implanted parental vs. Esc tumors. **i**–**k**, Kinetic analyses of cell growth *in silico* were performed by indicated tumor cell lines (*n* = 3 or 6). ** $P < 0.01$, *** $P < 0.0001$ by Student's *t* test (**c**,**e**). Data are representative of two independent experiments. Source data and exact *P* value are provided as a Source Data file.

Supplementary Fig. 4

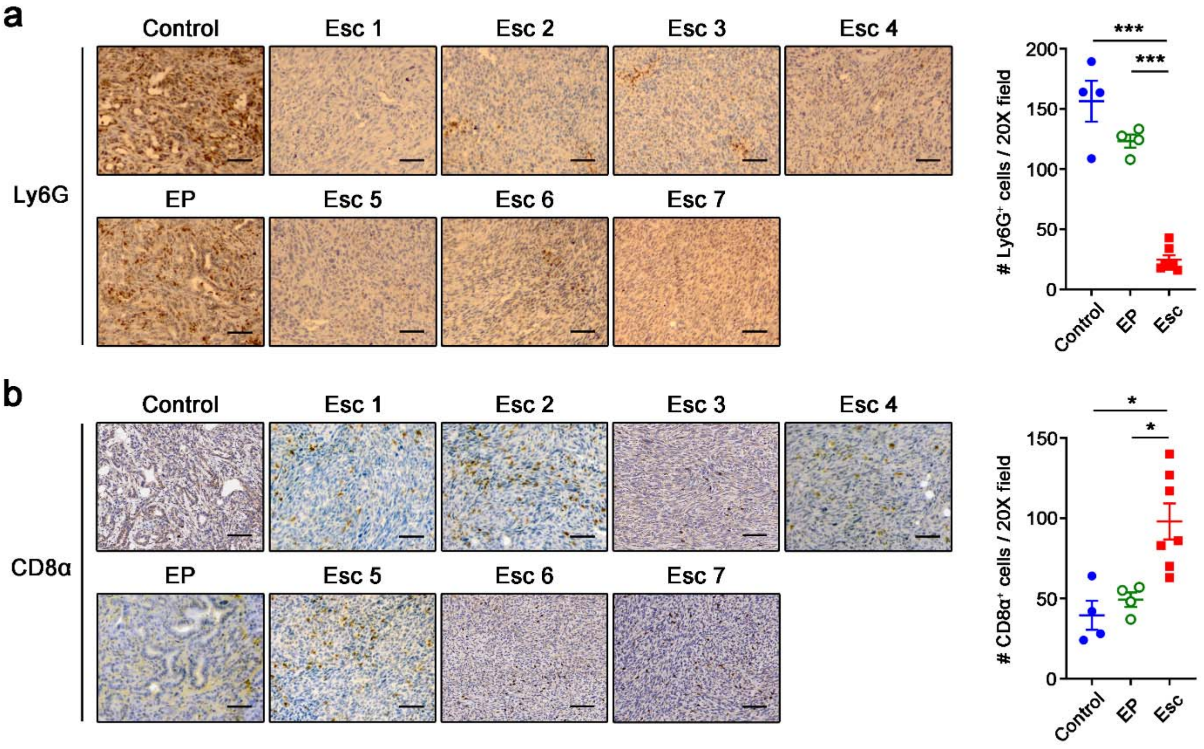

Supplementary Fig. 4 Original Esc tumors exhibit "hot" tumor phenotypes.

Representative IHC images of Ly6G (**a**) and CD8a (**b**) in the original primary tumor tissues from control, EP, and Esc tumor-bearing mice (n = 2 or 7). Mean counts of positive cells in 2-5 magnification fields per tissue section are depicted (right). Two control and EP lines for each were analyzed (duplicates). Each datapoint in the Esc plot represents the average results from an individual cell line. Scale bars, 250 μm. * $P < 0.05$, *** $P < 0.0001$ by Mann-Whitney t test. Source data and exact P value are provided as a Source Data file.

Supplementary Fig. 5

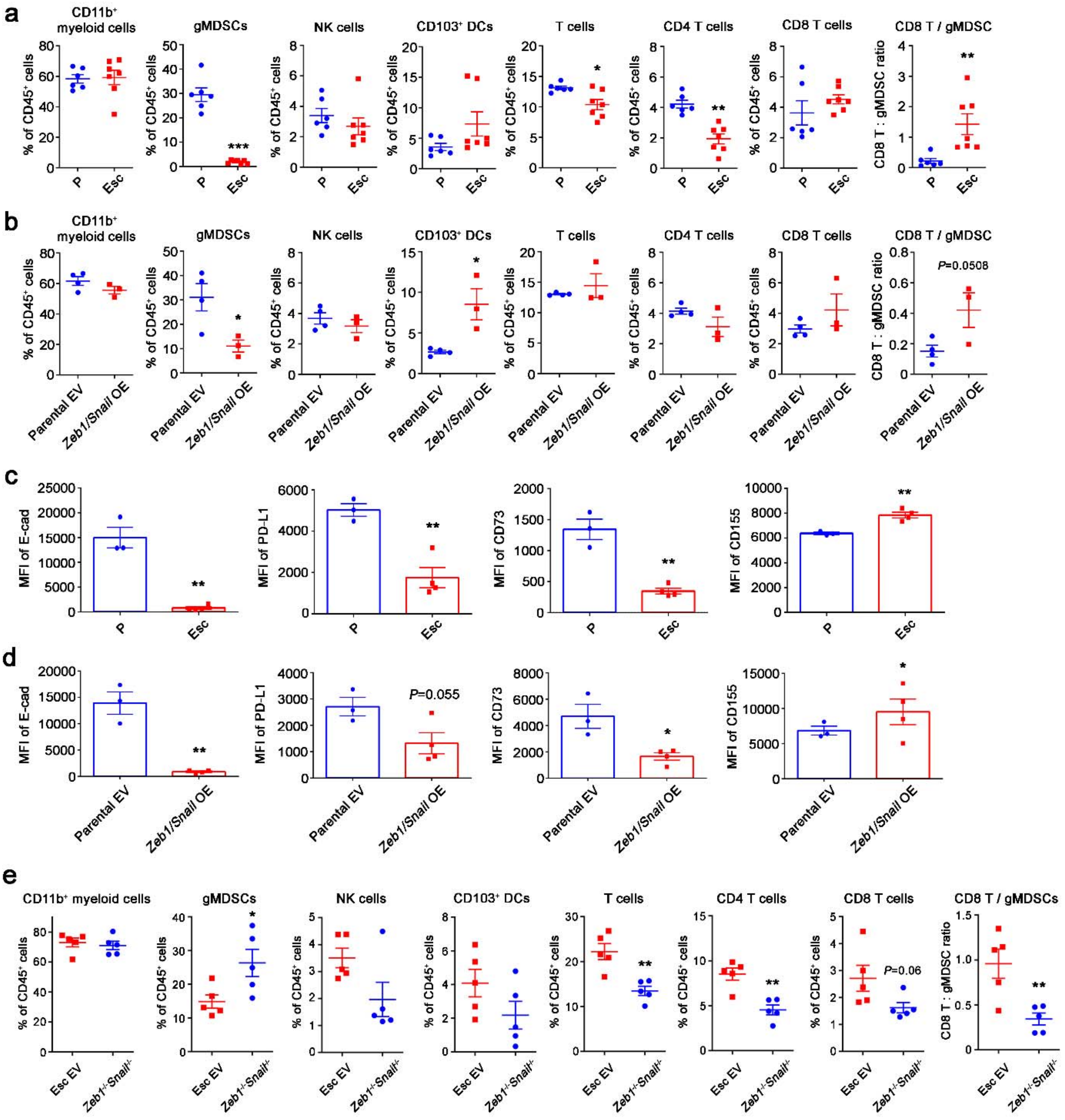

Supplementary Fig. 5 EMT-induced acquired immunotherapy resistance is not associated with an immunosuppressive TME. **a**,**b**, Flow cytometric analysis of immune populations in orthotopically implanted 4662 parental (P) vs. Esc (**a**) and 4662 parental EV vs. *Zeb1/Snail* OE tumors (**b**) on day 18 post the transplant. **c**,**d**, MFI of E-cadherin and co-inhibitory molecules PD-L1, CD73, and CD155 on parental vs. Esc tumors (**c**) and parental EV vs. *Zeb1/Snail* OE tumors (**d**). For assessing PD-L1 expression, cells were treated with IFN-γ (100 ng/ml) overnight. Each dot in all figures represents biological replicates. **e**, The frequencies of immune populations in s.c. implanted 4662 Esc EV and *Zeb1*$^{-/-}$*Snail*$^{-/-}$ tumors on day 18 post inoculation were analyzed by flow cytometry (n = 5). * $P < 0.05$, ** $P < 0.01$ by Student's t test. Data represent two independent experiments. Source data and exact P value are provided as a Source Data file.

Supplementary Fig. 6

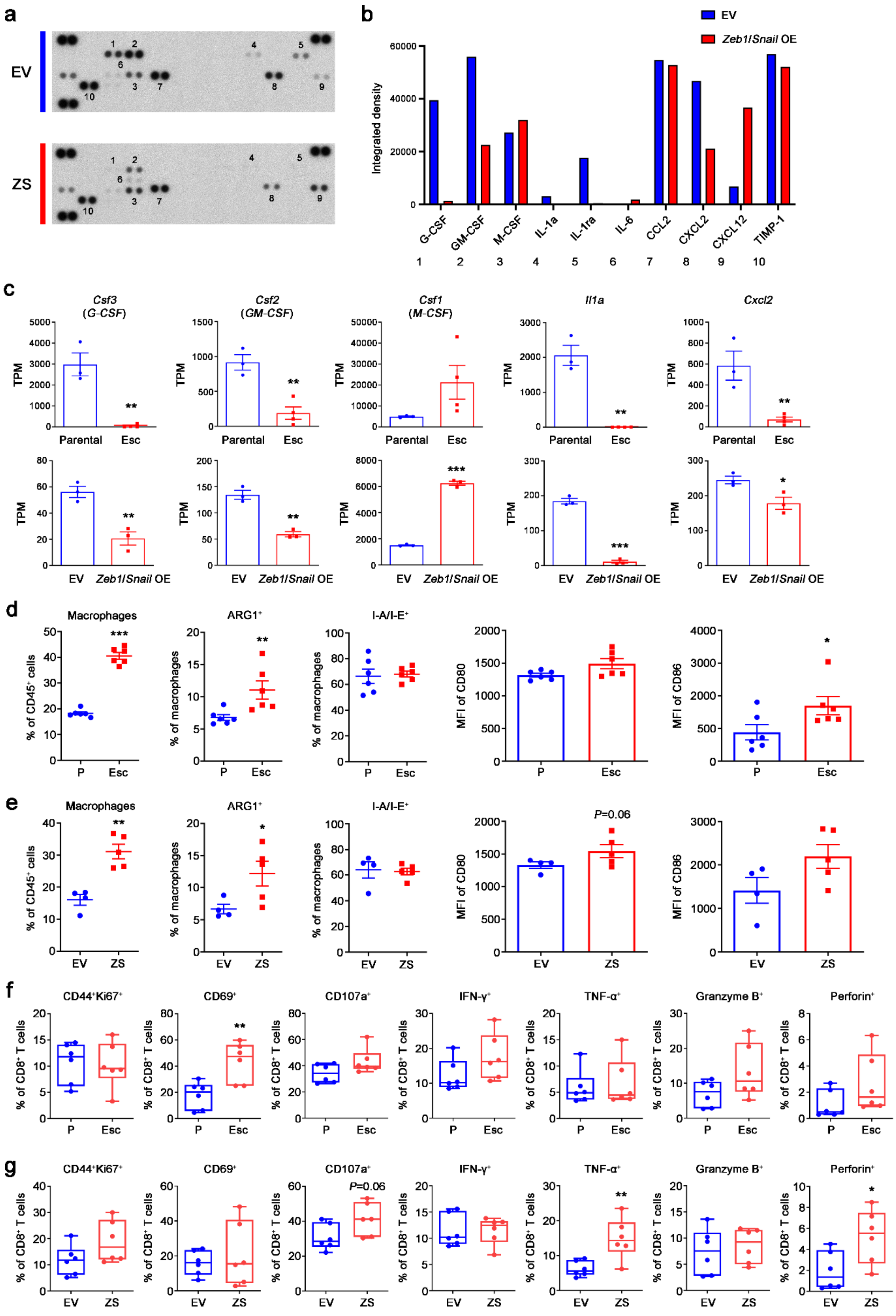

Supplementary Fig. 6 Esc and *Zeb1/Snail* OE tumor cells express lower levels of gMDSC recruiting cytokines and chemokines. **a**,**b**, Levels of the indicated factors measured by cytokine/chemokine array in tissue culture supernatants from 4662 EV and *Zeb1/Snail* OE (ZS) tumor cells. **c**, mRNA levels (TPM) of myeloid cell-recruiting cytokines and chemokines in 4662 parental vs. Esc (top) (n = 3 or 4) and 4662 parental EV vs. *Zeb1/Snail* OE (bottom) (n = 3) tumor cells. **d**,**e**, The frequencies of total, Arginase I^+, and MHC II^+ macrophages and MFI of CD80 and CD86 in macrophages in 4662 parental vs. Esc (**d**) (n = 6) and 4662 parental EV vs. *Zeb1/Snail* OE tumors (**e**) (n = 4 or 5). **f**,**g**, The frequencies of CD8 T cells expressing activation markers and effector molecules among total CD8 T cells in 4662 parental vs. Esc (**f**) (n = 6) and 4662 parental EV vs. *Zeb1/Snail* OE tumors (**g**) (n = 6) a week post immunotherapy were depicted. Each dot represents each biological replicate. * $P < 0.05$, ** $P < 0.01$, *** $P < 0.0001$ by Student's t test. Source data and exact P value are provided as a Source Data file.

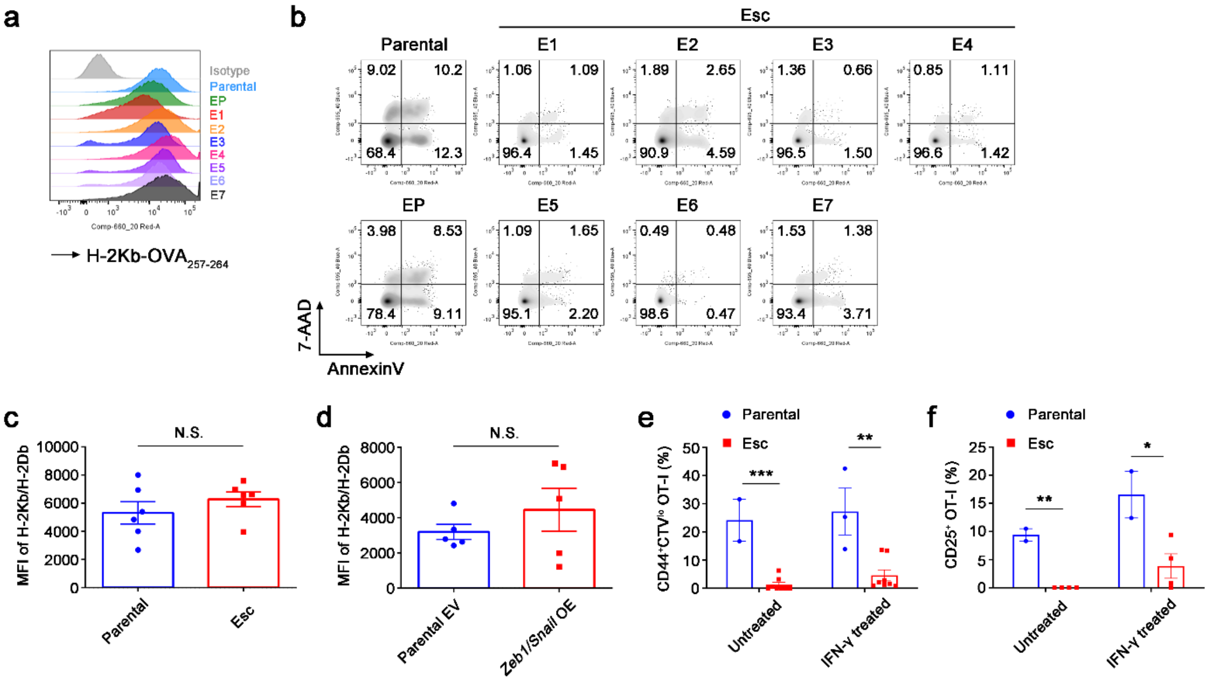

Supplementary Fig. 7 EMT-induced acquired immunotherapy resistance is not due to defects in MHC I expression and antigen presentation, but deregulated cytotoxic T cell activity. **a**, The expression of H-2Kb–$OVA_{257-264}$ in OVA-tdTomato^{+} 4662 parental, EP, and Esc lines treated with IFN-γ (100 ng/ml) for 1 d. **b**, The expression of AnnexinV and 7-AAD in OVA-tdTomato^{+} 4662 parental, EP, and Esc lines co-cultured with activated OT-I for 2 d. **c**,**d**, MFIs of H-2Kb/H-2Db in s.c. implanted YFP^{+} 4662 parental vs. Esc (**c**) and parental EV vs. *Zeb1*/*Snail* OE tumors (**d**) on day 18 post inoculation. **e**,**f**, Naïve OT-I cells labeled with CellTrace Violet (CTV) were primed by untreated or IFN-γ treated OVA^{+} parental and Esc tumors for 3 d. The frequencies of activated OT-I cells were presented by CTV dilution and CD44 (**e**) and CD25 (**f**) expression. Each dot in all figures represents biological replicates. N.S., non-significant. * $P < 0.05$, ** $P < 0.01$, *** $P < 0.0001$ by Student's t test. Data represent two independent experiments. Source data and exact P value are provided as a Source Data file.

Supplementary Fig. 8

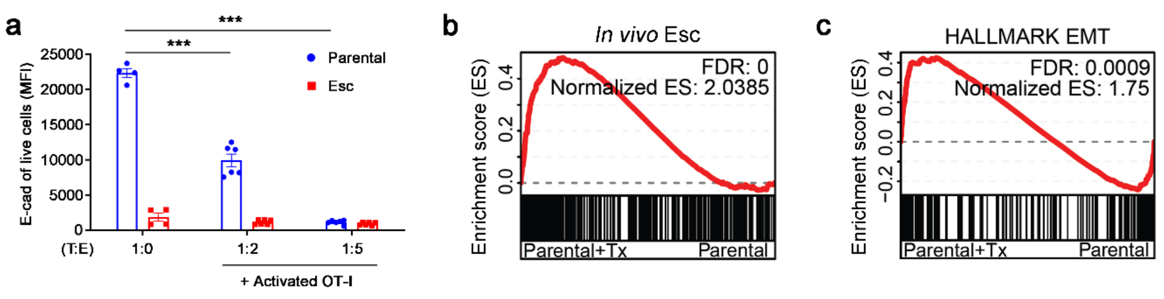

Supplementary Fig. 8 PDAC cell survival under immune pressure is associated with EMT. **a**, MFI of E-cadherin on surviving 4662 parental and Esc tumors with or without OT-I co-culture for 2 d, gated on AnnexinV^{-}7-AAD^{-}. Each dot represents biological replicates. **b**, GSEA plot of a gene signature derived from s.c. implanted Esc tumors in s.c. implanted clonal parental tumors with (+Tx) or without immunotherapy including agonistic anti-CD40 Ab, anti-CTLA-4 Ab, and anti-PD-1 Ab (FCP). Tumor cells were prepared 7 days after starting immunotherapy that was 3 weeks post tumor inoculation. **c**, GSEA plot of the EMT Hallmark in s.c. implanted clonal parental tumors with (+Tx) or without immunotherapy. *** $P < 0.0001$ by Student's *t* test. Source data are provided as a Source Data file.

Supplementary Fig. 9

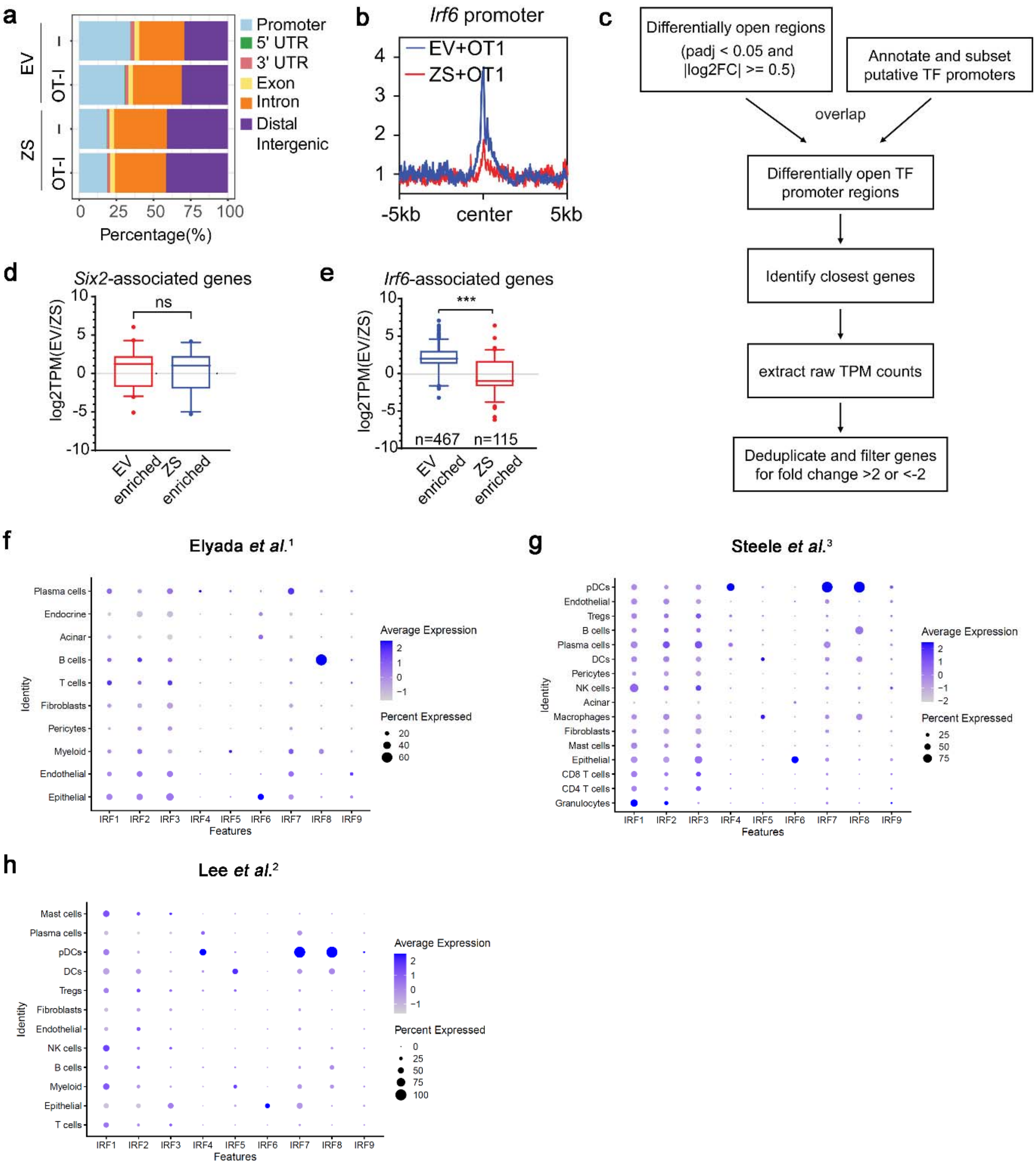

Supplementary Fig. 9 Chromatin accessibility and gene activity of IRF6 are repressed by *Zeb1* and *Snail* and its expression is exclusive to epithelial cells in human PDAC. **a**, The proportions of genomic regions with open chromatin status in clonal 4662 parental EV and *Zeb1*/*Snail* OE (ZS) tumors in the presence or absence of OT-I for 1 d. **b**, Aggregate plots comparing the average ATAC signal of EV (blue) and *Zeb1/Snail* OE (red) tumors with OT-I around all putative *Irf6* promoter sequences. **c**, Workflow to identify the expression levels of putative target genes based on genomic regions with differentially open chromatin in parental EV vs. *Zeb1/Snail* OE tumors. **d**,**e**, Boxplots of log2fold changes in the expression of *Six2*-associated genes (**d**) or *Irf6*-associated genes (**e**) with differentially open chromatin in parental EV vs. *Zeb1/Snail* OE tumors with OT-I. **f**–**h**, The expression levels and percent positive of each IRF family member among various cell types in human PDAC datasets are illustrated. ns, non-significant. *** $P < 0.0001$ by Student's t test.

Supplementary Fig. 10

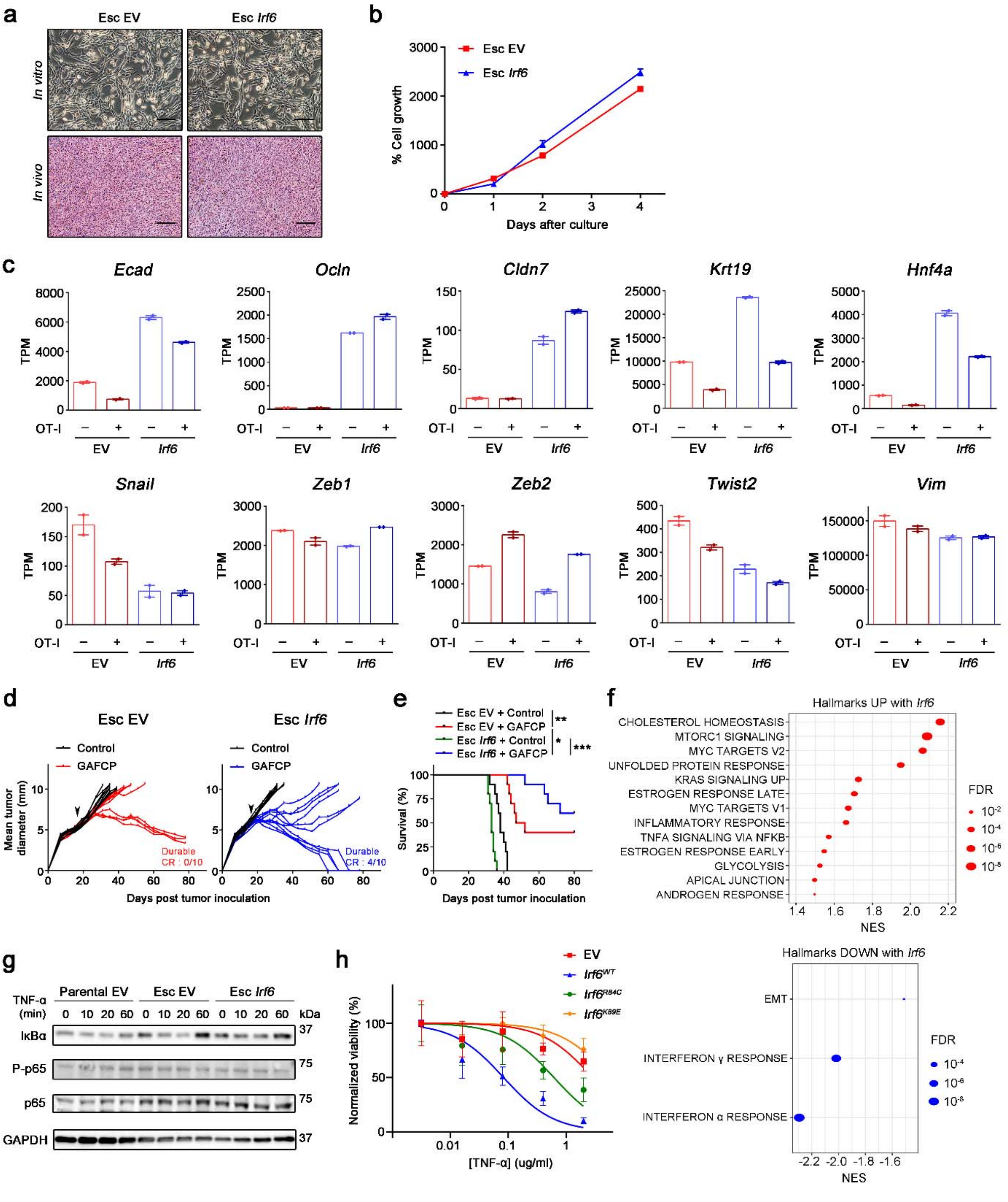

Supplementary Fig. 10 *In vitro* and *in vivo* phenotypes of *Irf6*-expressing Esc tumors. a, Representative bright field (top) and H&E (bottom) images of 4662 Esc EV and Esc *Irf6* PDAC from *in vitro* and s.c. implanted tumors, respectively. Scale bars, 250 μm. **b**, *In vitro* kinetics of cell growth of 4662 Esc EV and Esc *Irf6* tumors was analyzed. **c**, TPMs of epithelial (top) and mesenchymal (bottom) genes in 4662 Esc EV and Esc *Irf6* tumors with or without OT-I. Each dot represents biological replicates. **d**,**e**, Tumor growth (**d**) and survival (**e**) of mice bearing 4662 Esc EV and Esc *Irf6* tumors treated with control IgG or combination chemoimmunotherapy (GAFCP, arrow) (n = 10). **f**, GSEA of hallmark gene sets from the Molecular Signature Database summarized by FDR and NES. Hallmarks upregulated (top) and downregulated (bottom) in 4662 Esc *Irf6* vs. Esc EV tumors co-cultured with activated cognate CD8 T cells are shown. **g**, Immunoblots of NF-κB signaling pathway related molecules in 4662 parental EV, Esc EV, and Esc *Irf6* tumors treated with TNF-α for indicated time. **h**, Normalized viability of 4662 Esc EV and Esc tumors transduced with $Irf6^{WT}$, $Irf6^{R84C}$, or $Irf6^{K89E}$ and treated with varying concentrations of TNF-α in the presence of IFN-γ plus cycloheximide for 48 h. * $P < 0.05$, ** $P < 0.01$, *** $P < 0.0001$ by log-rank (Mantel-Cox) test. Data represent two independent experiments. Source data and exact P value are provided as a Source Data file.

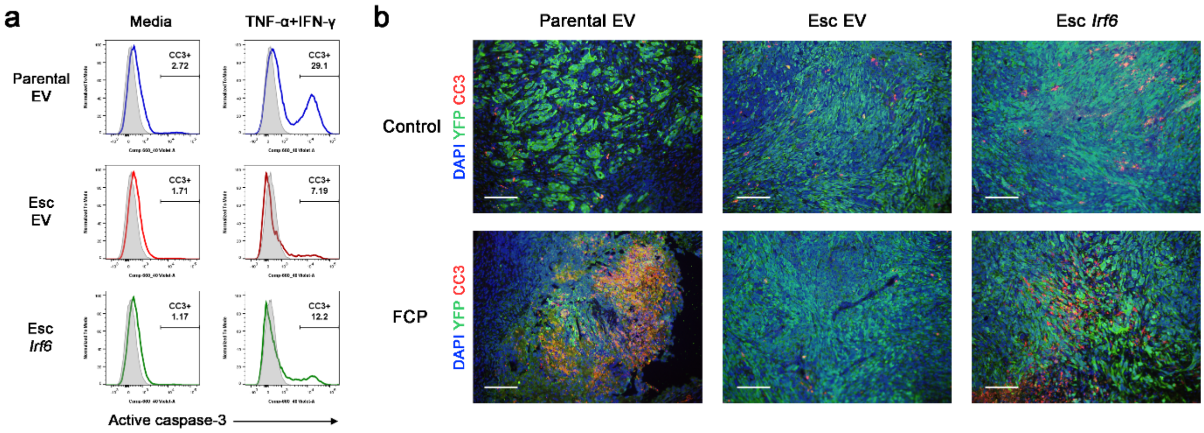

Supplementary Fig. 11 Irf6 re-expression increases cleaved caspase-3 in Esc cells upon pro-apoptotic stimulation. a, Flow cytometry of active caspase-3 in 4662 parental EV, Esc EV, and Esc *Irf6* tumors treated with or without TNF-α (0.5 μg/ml) plus IFN-γ (0.2 μg/ml) in the presence of cycloheximide (1 μg/ml) for 48 h. **b**, Representative IF staining images of cleaved caspase-3 in 4662 parental EV, Esc EV, and Esc *Irf6* tumors a week post control IgG or immunotherapy (FCP). Scale bars, 250 μm. Data represent two independent experiments.

Supplementary Fig. 12

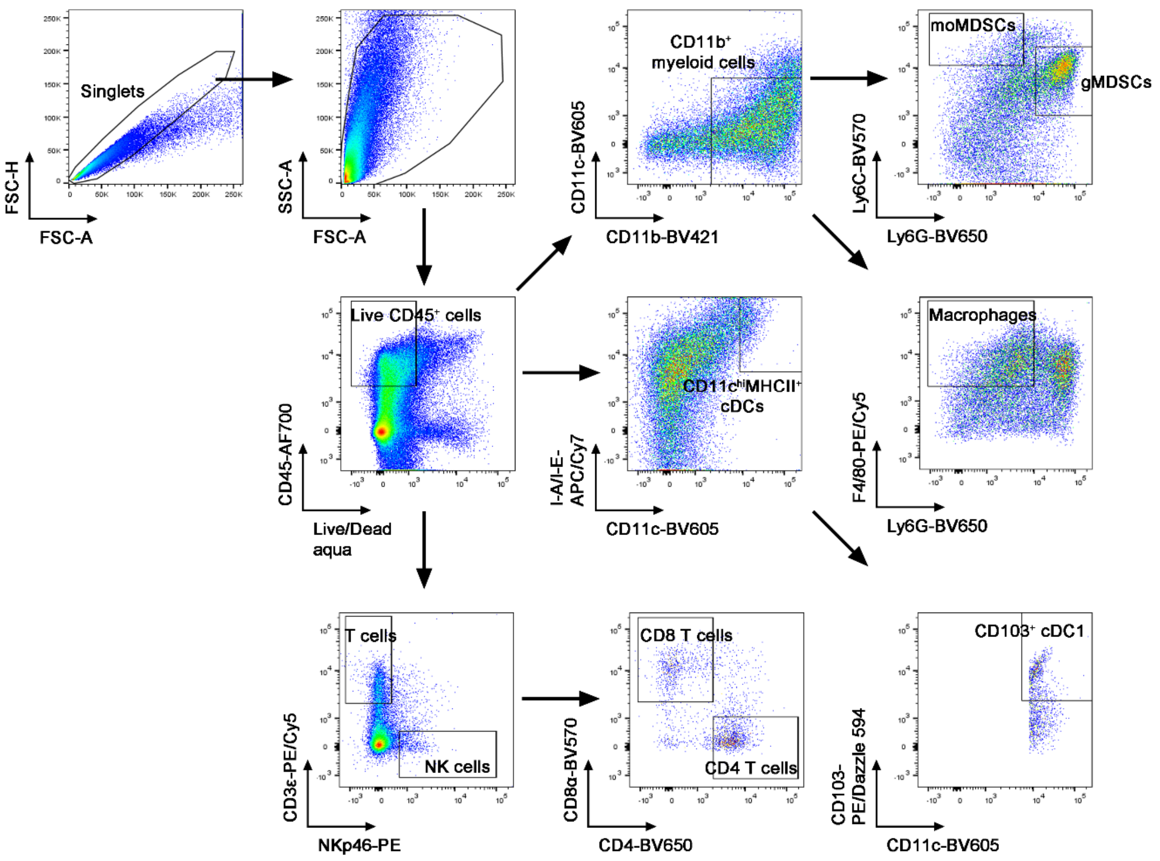

Supplementary Fig. 12 Gating strategy for assessing tumor-infiltrating immune populations, related to **Figure 3** and **Supplementary Fig. 5&6**

▼ Supplementary Fig. 10g: P-p65

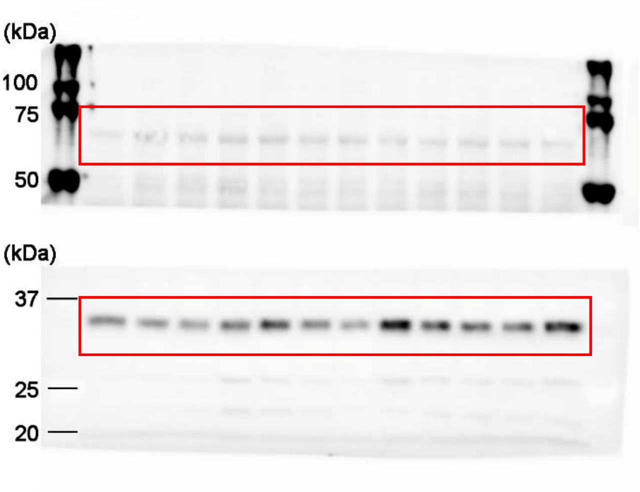

▲ Supplementary Fig. 10g: IκBα

▼ Supplementary Fig. 10g: p65

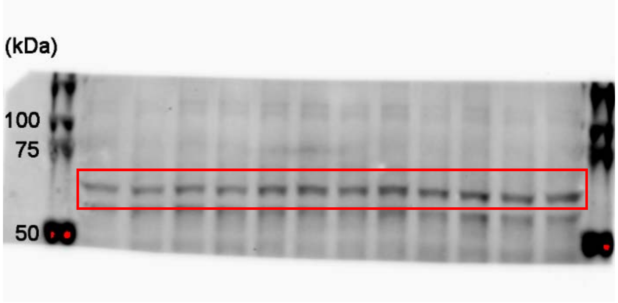

▼ Supplementary Fig. 10g: GAPDH

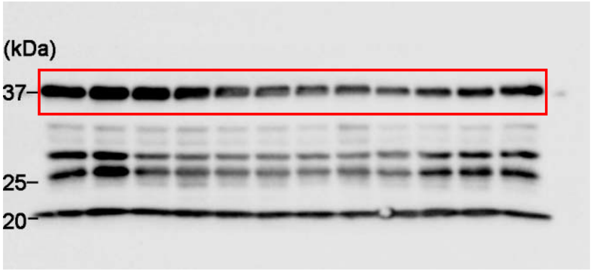

Supplementary Fig. 13 Unprocessed western blots. Western blot ChemiDoc (Bio-Rad) images. Boxes indicate lanes shown in the figures.

Supplementary Table 1. The list of candidate genes by GSEA and metascape analysis

Biological pathways	Genes
Cellular response to external stimulus including growth factor	Ccn4, Egr3, Hgf, Trps1, Lrp1, Bmp8b, Hpgd
Regulation of phospholipase activity	Egr3, Hgf, Antxr1, Lrp1, Fzd3
Mesenchymal cell proliferation	Ccn4, Fzd3
Signaling by receptor tyrosine kinases	Egr2, Hgf, Esrp1, Ppp2r2c, Spint1, Tnk1, Prkcz, Esrp2
Negative regulation of cell population proliferation	Irf6, Il1a, Spint1, Cbfa2t3, Cd24a
TNFα signaling via NFκB	Il1a, Bhlhe40, Fos, Atf3, Phlda2
Interferon alpha response	Samd9l, Parp9, Parp14, Irf7
Interferon gamma response	Samd9l, Parp14, Irf7, Oas2, Pml, Zbp1
Inflammatory response	Trim15, Il1a, Pglyrp1, Prkcz, Unc13d, Cd24a, Irf7, Tlr3
IL2 STAT5 signaling	Irf6, Dhrs3, Cish, Bhlhe40
p53 pathway	Il1a, Osgin1, Perp, Fos, Atf3
Estrogen response early	Dhrs3, Cish, Pmaip1, Esrp2, Cbfa2t3, Bhlhe40, Fos
3-phosphoinositide biosynthesis	Ppp2r2c, Inpp5j, Ptprh
Molecular mechanisms of cancer	Lrp1, Bmp8b, Prkcz

Supplementary Table 2. The sequences of primers and CRISPR sgRNAs

Target	Sequence	Supplier
Primer: mCdh1 Forward	CTCCAGTCATAGGGAGCTGTC	IDT
Primer: mCdh1 Reverse	TCTTCTGAGACCTGGGTACAC	IDT
Primer: mCdh2 Forward	AGTGGCAGGTAGCTGTAAAC	IDT
Primer: mCdh2 Reverse	TGGCAAGTTGTCTAGGGAATAC	IDT
Primer: mVim Forward	CCCTGAACCTGAGAGAAACTAAC	IDT
Primer: mVim Reverse	CTCTGGTCTCAACCGTCTTAATC	IDT
Primer: mSnail Forward	GTCTCAGAAGGGACCATGAATAA	IDT
Primer: mSnail Reverse	ATAGTTCTGGGAGACACATTGG	IDT
Primer: mTwist1 Forward	GGACAAGCTGAGCAAGATTCA	IDT
Primer: mTwist1 Reverse	CGGAGAAGGCGTAGCTGAG	IDT
Primer: mZeb1 Forward	TCGGAAGACAGAGAATGGAATG	IDT
Primer: mZeb1 Reverse	CCTCTTACCTGTGTGCTCATATT	IDT
Primer: mTbp Forward	AGAACAATCCAGACTAGCAGCA	IDT
Primer: mTbp Reverse	GGGAACTTCACATCACAGCTC	IDT
sgRNA: Zeb1 #1	CATTTATCCTGAGGCGCCCG	IDT
sgRNA: Zeb1 #2	AGCTTGAACGTCATATGACA	IDT
sgRNA: Zeb1 #3	CCCGCAGGGTTACTCTTGTG	IDT
sgRNA: Zeb1 #4	CCTTTAAAGAACCTTCTGTC	IDT
sgRNA: Snail #1	CTCTCCTGGTACCCCAAGTG	IDT
sgRNA: Snail #2	TGGCCAAGGACCCCCAGTCG	IDT
sgRNA: Snail #3	CGCTGTCCGATGAGGACAGT	IDT
sgRNA: Snail #4	CTTCCAGCAGCCCTACGACC	IDT
sgRNA: Irf6 #1	TCCTGAACATCAACGGTGAG	IDT
sgRNA: Irf6 #2	CAAATTTCAGTATCGTGGGA	IDT
sgRNA: Irf6 #3	TGTGACATCCCCCAGACCCA	IDT
sgRNA: Irf6 #4	CTGGAAACATGCCACGCGGC	IDT
sgRNA: Tradd #1;	AAACTGACGTGTGACTGCAC	IDT
sgRNA: Tradd #2	GACCGAGGAGAAACCACTGC	IDT
sgRNA: Tradd #3	GATCCTGTCTGAAGCCTACA	IDT
sgRNA: Tradd #4	CCTCCAAGCCTACCGCGAGG	IDT

sgRNA: Fadd #1	CGCTGCGCCGACACGATCTA	IDT
sgRNA: Fadd #2	ACATTGTGTGTGACAATGTG	IDT
sgRNA: Fadd #3	GGCCAAGATGGATGGGATTG	IDT
sgRNA: Fadd #4	AAGCTGGAGCGCGTGCAGAG	IDT
sgRNA: Casp8 #1	CTTCCTAGACTGCAACCGAG	IDT
sgRNA: Casp8 #2	CGGGGATACTGTCTGATCAT	IDT
sgRNA: Casp8 #3	CTACATCCCACACAAGAAGC	IDT
sgRNA: Casp8 #4	AGGTCAACAAGAGCCTGCTG	IDT
Primer: mIrf6^{R84C} Forward	GGCTCAGCTCTGCTGTGCTCT	IDT
Primer: mIrf6^{R84C} Reverse	TTCCATTTAGCTGGGTCAGGATC	IDT
Primer: mIrf6^{K89E} Forward	TGCTCTCAACGAAAGCAGGGAG	IDT
Primer: mIrf6^{K89E} Reverse	CAGCGGAGCTGAGCCTTC	IDT
Primer: mIrf6 promoter#1 Forward	CTGTGGCATCCAGGGCTAGG	IDT
Primer: mIrf6 promoter#1 Reverse	CTAGGTGCGGCTGGGAAC	IDT
Primer: mIrf6 promoter#2 Forward	CCAGCCGCACCTAGCC	IDT
Primer: mIrf6 promoter#2 Reverse	CTCAGGAGCAGGTGCACAA	IDT
Primer: mIrf6 exon 6 Forward	GTATCGTGGGAAGGAGTATGGG	IDT
Primer: mIrf6 exon 6 Reverse	CAGGTCCCCATAGAAGAGCC	IDT

References

1. Elyada E, *et al.* Cross-Species Single-Cell Analysis of Pancreatic Ductal Adenocarcinoma Reveals Antigen-Presenting Cancer-Associated Fibroblasts. *Cancer discovery* **9**, 1102-1123 (2019).
2. Lee JJ, *et al.* Elucidation of Tumor-Stromal Heterogeneity and the Ligand-Receptor Interactome by Single-Cell Transcriptomics in Real-world Pancreatic Cancer Biopsies. *Clinical cancer research : an official journal of the American Association for Cancer Research* **27**, 5912-5921 (2021).
3. Steele NG, *et al.* Multimodal Mapping of the Tumor and Peripheral Blood Immune Landscape in Human Pancreatic Cancer. *Nature cancer* **1**, 1097-1112 (2020).
