# Supplementary material for: Plasticity-induced repression of Irf6 underlies acquired resistance to cancer immunotherapy in pancreatic ductal adenocarcinoma
Source: Nat Commun. 2024 Feb 20;15:1532. doi: 10.1038/s41467-024-46048-7 (PMC10879147; doi:10.1038/s41467-024-46048-7)
Supplement: Supplementary file 3 — Description of Additional Supplementary Files [file 41467_2024_46048_MOESM3_ESM.pdf]

Description of Additional Supplementary Information

Title: Supplementary Data 1

Description: Differentially expressed gene sets in *Zeb1/Snail* OE compared to EV. Genes with log2(fold change) > 1.5 or <-1.5 and adjusted p value < 0.01 by RNA-seq analysis were listed.

Title: Supplementary Data 2

Description: *Irf6* signature gene sets. This signature was obtained from top ranked genes by a STAT value following the DESeq2 analysis of *Irf6* re-expressing Esc vs. EV Esc tumor cells.
