# Supplementary material for: Plasticity-induced repression of Irf6 underlies acquired resistance to cancer immunotherapy in pancreatic ductal adenocarcinoma
Source: Nat Commun. 2024 Feb 20;15:1532. doi: 10.1038/s41467-024-46048-7 (PMC10879147; doi:10.1038/s41467-024-46048-7)
Supplement: Supplementary file 6 — Reporting Summary [file 41467_2024_46048_MOESM6_ESM.pdf]

nature portfolio

Corresponding author(s): Ben Z. Stanger
Last updated by author(s): Jan 30, 2024

Reporting Summary

Nature Portfolio wishes to improve the reproducibility of the work that we publish. This form provides structure for consistency and transparency in reporting. For further information on Nature Portfolio policies, see our Editorial Policies and the Editorial Policy Checklist.

Statistics

For all statistical analyses, confirm that the following items are present in the figure legend, table legend, main text, or Methods section.

n/a	Confirmed	
☐	☒	The exact sample size (*n*) for each experimental group/condition, given as a discrete number and unit of measurement
☐	☒	A statement on whether measurements were taken from distinct samples or whether the same sample was measured repeatedly
☐	☒	The statistical test(s) used AND whether they are one- or two-sided<br>*Only common tests should be described solely by name; describe more complex techniques in the Methods section.*
☒	☐	A description of all covariates tested
☐	☒	A description of any assumptions or corrections, such as tests of normality and adjustment for multiple comparisons
☐	☒	A full description of the statistical parameters including central tendency (e.g. means) or other basic estimates (e.g. regression coefficient) AND variation (e.g. standard deviation) or associated estimates of uncertainty (e.g. confidence intervals)
☐	☒	For null hypothesis testing, the test statistic (e.g. *F*, *t*, *r*) with confidence intervals, effect sizes, degrees of freedom and *P* value noted<br>*Give P values as exact values whenever suitable.*
☒	☐	For Bayesian analysis, information on the choice of priors and Markov chain Monte Carlo settings
☒	☐	For hierarchical and complex designs, identification of the appropriate level for tests and full reporting of outcomes
☒	☐	Estimates of effect sizes (e.g. Cohen's *d*, Pearson's *r*), indicating how they were calculated

Our web collection on statistics for biologists contains articles on many of the points above.

Software and code

Policy information about availability of computer code

Data collection	Flow cytometry analysis was performed using LSR II and software FACSDiva v8.0 (BD Biosciences).<br>qPCR analysis was performed using CFX384 Real-Time System and software BioRadCFXManager (BioRad).<br>RNA-seq and ATAC-seq analyses were performed using Illumina next generation sequencers and software NextSeq System Suite.<br>Tissue image acquisition was performed using Olympus BX41 microscope with a DP25 camera and Olympus IX71 inverted multicolor fluorescent microscope with a DP71 camera.<br>Cell proliferation and viability assay were performed using SpectraMax M5 (Molecular Devices) and Synergy HTX (BioTek) microplate readers.<br>Western blot images were acquired using ChemiDoc (BioRad).
Data analysis	FlowJo v10.8.1 (Tree Star), Prism v9.3.1 (GraphPad)<br>GSEA v4.3.0 (Broad Institute) https://www.gsea-msigdb.org/gsea/index.jsp<br>IGV genome browser v2.14.1 (Robinson et al., 2011) https://software.broadinstitute.org/software/igv/<br>ImageJ Fiji v2.3.0 (NIH) https://imagej.nih.gov/ij/<br>R v4.1.2 (The R Foundation) https://cran.r-project.org/<br>Salmon v1.8.0 (Dobin et al., 2013) https://combine-lab.github.io/salmon/<br>DESeq2 Bioconductor v3.14 (Love et al., 2014) https://bioconductor.org/packages/release/bioc/html/DESeq2.html<br>HOMER v4.11 (Heinz et al., 2010) http://homer.ucsd.edu/homer/<br>Cutadapt v3.5 (Martin, 2011) https://cutadapt.readthedocs.io/en/v3.5/<br>Bowtie2 v2.4.4 (Langmead and Salzberg, 2012) https://bowtie-bio.sourceforge.net/bowtie2/index.shtml<br>Sambamba v0.7.1 (Tarasov et al., 2015) https://github.com/biod/sambamba

SAMtools v1.9 (Li et al., 2009) http://samtools.sourceforge.net
Genrich v0.6.1 https://github.com/jsh58/Genrich
BEDtools v2.30.0 (Quinlan and Hall, 2010) https://bedtools.readthedocs.io/en/latest/
FeatureCounts v2.0.1 (Liao et al., 2014) https://subread.sourceforge.net/featureCounts.html
ChIPseeker v1.32.1 (Yu et al., 2015) https://rdrr.io/bioc/ChIPseeker/f/vignettes/ChIPseeker.Rmd
GREAT (McLean et al., 2010) http://great.stanford.edu/
BEDOPS (Neph et al., 2012) https://github.com/bedops
DeepTools v3.5.1 (Ramirez et al., 2016) https://deeptools.readthedocs.io/en/develop/
Seurat v4 (Hao et al., 2021) https://satijalab.org/seurat/

For manuscripts utilizing custom algorithms or software that are central to the research but not yet described in published literature, software must be made available to editors and reviewers. We strongly encourage code deposition in a community repository (e.g. GitHub). See the Nature Portfolio guidelines for submitting code & software for further information.

Data

Policy information about availability of data

All manuscripts must include a data availability statement. This statement should provide the following information, where applicable:

- Accession codes, unique identifiers, or web links for publicly available datasets
- A description of any restrictions on data availability
- For clinical datasets or third party data, please ensure that the statement adheres to our policy

NGS datasets generated in this study are available in the NCBI Gene Expression Omnibus (GEO) under accession number GSE222225 (SuperSeries) with SubSeries accession number GSE222223 (ATAC-seq) and GSE222224 (RNA-seq). Reviewers can access the SuperSeries with following token: sdqxgaokljirvcj

Research involving human participants, their data, or biological material

Policy information about studies with human participants or human data. See also policy information about sex, gender (identity/presentation), and sexual orientation and race, ethnicity and racism.

Reporting on sex and gender	N/A
Reporting on race, ethnicity, or other socially relevant groupings	N/A
Population characteristics	N/A
Recruitment	N/A
Ethics oversight	N/A

Note that full information on the approval of the study protocol must also be provided in the manuscript.

Field-specific reporting

Please select the one below that is the best fit for your research. If you are not sure, read the appropriate sections before making your selection.

☒ Life sciences ☐ Behavioural & social sciences ☐ Ecological, evolutionary & environmental sciences

For a reference copy of the document with all sections, see nature.com/documents/nr-reporting-summary-flat.pdf

Life sciences study design

All studies must disclose on these points even when the disclosure is negative.

Sample size	No statistical method was used to pre-determine the sample size, which was chosen on the basis of previous experimental experience. If any, it was based on the Resource Equation method (Charan J and Kantharia ND. J Pharmacol Pharmcother. 2013 Oct;4(4):303-6).
Data exclusions	No data were excluded from the analyses.
Replication	All experimental replication was attempted at least two times or two biological replicates for NGS analyses. The number of biological replicates were provided in the figure legends.
Randomization	Mice bearing PDAC were randomly allocated to each group. For in vitro experiments, samples were randomly assigned to control and experimental groups.
Blinding	For measuring tumor phenotypes in vivo, investigators were not blinded to group information, however, the results were reproducible by two independent researchers in different animal facilities. For in vitro experiments, blinding was not required because all the samples were analyzed in a consistent manner.

Reporting for specific materials, systems and methods

We require information from authors about some types of materials, experimental systems and methods used in many studies. Here, indicate whether each material, system or method listed is relevant to your study. If you are not sure if a list item applies to your research, read the appropriate section before selecting a response.

Materials & experimental systems

n/a	Involved in the study
☐	☒ Antibodies
☐	☒ Eukaryotic cell lines
☒	☐ Palaeontology and archaeology
☐	☒ Animals and other organisms
☒	☐ Clinical data
☒	☐ Dual use research of concern
☒	☐ Plants

Methods

n/a	Involved in the study
☒	☐ ChIP-seq
☐	☒ Flow cytometry
☒	☐ MRI-based neuroimaging

Antibodies

Antibodies used

InVivoMAb anti-mouse PD-1 (CD279) (clone RMP1-14), Bio X Cell #BE0146; RRID:AB_10949053
InVivoMAb anti-mouse CTLA-4 (CD152) (clone 9H10), Bio X Cell #BE0131; RRID:AB_10950184
InVivoMAb anti-mouse CD40 (clone FGK45), Bio X Cell #BE0016-2; RRID:AB_1107647
InVivoMAb anti-mouse CD4 (clone GK1.5), Bio X Cell #BE0003-1; RRID:AB_1107636
InVivoMAb anti-mouse CD8α (clone 2.43), Bio X Cell #BE0061; RRID:AB_1125541
InVivoMAb anti-mouse NK1.1 (clone PK136), Bio X Cell #BE0036; RRID:AB_1107737
InVivoMAb rat IgG2a isotype control (clone 2A3), Bio X Cell #BE0089; RRID:AB_1107769
InVivoMAb rat IgG2b isotype control (clone LTF-2), Bio X Cell #BE0090; RRID:AB_1107780
InVivoMAb polyclonal Syrian hamster IgG, Bio X Cell #BE0087; RRID:AB_1107782
InVivoMAb mouse IgG2a isotype control (clone C1.18.4), Bio X Cell #BE0085; RRID:AB_1107771
Anti-mouse CD335 (NKp46) (clone 29A1.4), PE, BioLegend #137604 (1:100 for FACS); RRID:AB_2235755
Anti-mouse CD103 (clone 2E7), PE/Dazzle 594, BioLegend #121430 (1:100 for FACS); RRID:AB_2566493
Anti-mouse H-2Kb/H-2Db (clone 28-8-6), PE/Cy7, BioLegend #114616 (1:100 for FACS); RRID:AB_2750194
Anti-mouse H-2Kb bound to SIINFEKL (clone 25-D1.16), APC, BioLegend #141606 (1:100 for FACS); RRID:AB_11219595
Anti-mouse F4/80 (clone BM8), PE/Cy5, BioLegend #123112 (1:100 for FACS); RRID:AB_893482
Anti-mouse CD45 (clone 30-F11), AF700, BioLegend #103128 (1:100 for FACS); RRID:AB_493715
Anti-mouse I-A/I-E (clone M5/114.15.2), APC/Cy7, BioLegend #107627 (1:100 for FACS); RRID:AB_1659252
Anti-mouse/human CD11b (clone M1/70), BV421, BioLegend #101251 (1:100 for FACS); RRID:AB_2562904
Anti-mouse Ly6C (clone HK1.4), BV570, BioLegend #128030 (1:100 for FACS); RRID:AB_2562617
Anti-mouse Ly6G (clone 1A8), BV650, BioLegend #127641 (1:100 for FACS); RRID:AB_2565881
Anti-mouse CD11c (clone N418), BV605, BioLegend #117334 (1:100 for FACS); RRID:AB_2562415
Anti-mouse CD3ε (clone 145-2C11), PE/Cy5, BioLegend #100310 (1:100 for FACS); RRID:AB_312675
Anti-mouse CD8α (clone 53-6.7), BV570, BioLegend #100740 (1:100 for FACS); RRID:AB_2563055
Anti-mouse CD4 (clone HK1.4), BV650, BioLegend #128030 (1:100 for FACS); RRID:AB_2562617
Anti-mouse CD25 (clone PC61), FITC, BioLegend #102005 (1:100 for FACS); RRID:AB_312854
Anti-mouse CD62L (clone MEL-14), APC/Cy7, BioLegend #104428 (1:100 for FACS); RRID:AB_830799
Anti-mouse CD44 (clone IM7), BV605, BD Biosciences #563058 (1:100 for FACS); RRID:AB_2737979
Anti-mouse/human CD324 (E-Cadherin) (clone DECMA-1), PE/Dazzle 594, BioLegend #147316 (1:100 for FACS); RRID:AB_2750304
Anti-mouse CD73 (clone TY/11.8), PerCP/Cy5.5, BioLegend #127214 (1:100 for FACS); RRID:AB_11219403
Anti-mouse CD274 (B7-H1, PD-L1) (clone 10F.9G2), PE/Cy7, BioLegend #124314 (1:100 for FACS); RRID:AB_10643573
Anti-mouse CD155 (PVR) (clone TX56), BV605, BioLegend #131519 (1:100 for FACS); RRID:AB_2716160
Anti-mouse CD95 (Fas) (clone SA367H8), PE, BioLegend #152607 (1:100 for FACS); RRID:AB_2632903
Anti-mouse/human Arginase 1 (clone A1exF5), PE, eBioscience #12-3697-80 (1:100 for FACS); RRID:AB_2734838
Anti-mouse CD80 (clone 16-10A1), PE/Dazzle 594, BioLegend #104737 (1:100 for FACS); RRID:AB_2564174
Anti-mouse CD86 (clone GL-1), BV605, BioLegend #105037 (1:100 for FACS); RRID:AB_11204429
Anti-mouse Ki-67 (clone 16A8), PE/Dazzle 594, BioLegend #652427 (1:100 for FACS); RRID:AB_2632695
Anti-mouse CD69 (clone H1.2F3), PE/Cy7, BioLegend #104512 (1:100 for FACS); RRID:AB_493564
Anti-mouse CD107a (LAMP-1) (clone 1D4B), FITC, BioLegend #121606 (1 ug/ml for FACS); RRID:AB_572007
Anti-mouse IFN-g (clone XMG1.2), PerCP/Cy5.5, BioLegend #505822 (1:100 for FACS); RRID:AB_961359
Anti-mouse TNFa (clone MP6-XT22), PE/Cy7, BioLegend #506324 (1:100 for FACS); RRID:AB_2256076
Anti-mouse/human Granzyme B (clone GB11), Pacific Blue, BioLegend #515408 (1 ug/ml for FACS); RRID:AB_2562196
Anti-mouse Perforin (clone S16009A), APC, BioLegend #154303 (1:100 for FACS); RRID:AB_2721462
Rabbit anti-active Caspase-3 (clone C92-605), BV650, BD Biosciences #564096 (1:20 for FACS); RRID:AB_2738589
Rabbit polyclonal anti-IκBα, Cell Signaling Technology #9242 (1:1000 for western); RRID:AB_331623
Rabbit polyclonal anti-phospho-NF-κB p65 (Ser536), Cell Signaling Technology #3031 (1:1000 for western); RRID:AB_330559
Rabbit monoclonal anti-NF-κB p65 (clone D14E12), Cell Signaling Technology #8242 (1:1000 for western); RRID:AB_10859369

Rabbit monoclonal anti-GAPDH (clone 14C10), Cell Signaling Technology #2118 (1:1000 for western); RRID:AB_561053
Rabbit polyclonal anti-TRADD, Cell Signaling Technology #3694 (1:1000 for western); RRID:AB_2209044
Mouse monoclonal anti-FADD (clone 1F7), Enzo Life Sciences #ADI-AAM-212-E (1 ug/ml for western); RRID:AB_2039114
Rabbi polyclonal anti-cleaved Caspase-3 (Asp175), Cell Signaling Technology #9661 (1:1000 for western, 1:200 for IF); RRID:AB_2341188
Rabbit monoclonal anti-Caspase-8 (clone D35G2), Cell Signaling Technology #4790 (1:1000 for western); RRID:AB_10545768
Chicken polyclonal anti-GFP, Abcam #ab13970 (1:200 for IF); RRID:AB_300798
Goat polyclonal anti-IRF6, Novus Biologicals #NBP1-51911 (1:1000 for western, 1:200 for IHC); RRID:AB_11003608
Rat monoclonal anti-mouse E-cadherin (clone ECCD-2), Takara Bio #M108 (1:200 for IHC); RRID:AB_2895157
Rabbit anti-Vimentin (clone D21H3), Cell Signaling Technology #5741 (1:200 for IHC); RRID:AB_10695459
Rabbit polyclonal anti-Twist1/2, GeneTex #GTX127310 (1:200 for IHC); RRID:AB_2885640
Rat anti-mouse Ly6G (clone 1A8), Biotin, Stem Cell Technologies #60031BT.1 (1:200 for IHC); RRID:AB_2877150
Rabbit anti-mouse CD8a (clone D4W2Z), Cell Signaling Technology #98941 (1:100 for IHC); RRID:AB_2756376
Rabbit polyclonal anti-Zeb1, Proteintech #21544-1-AP (4 ug for ChIP); RRID:AB_10734325
Rabbit polyclonal anti-SNAIL, Proteintech #13099-1-AP (4 ug for ChIP); RRID:AB_2191756
Ultra-LEAF purified anti-mouse TNF-α (clone MP6-XT22), BioLegend #506331 (5 ug/ml for neutralization); RRID:AB_11147367
Ultra-LEAF purified anti-mouse CD3ε (clone 145-2C11), BioLegend #100340 (2 ug/ml for T-cell activation); RRID:AB_11149115
Ultra-LEAF purified anti-mouse CD28 (clone 37.51), BioLegend #102116 (1 ug/ml for T-cell activation); RRID:AB_11147170

Validation

All the antibodies are commercially available and validated by manufacturers for the indicated purpose. Additional validation and relevant citations were provided in a RRID portal with the indicated RRID number. Depletions of specific immune population were confirmed by peripheral blood samples and end-of-study flow cytometry.

Eukaryotic cell lines

Policy information about cell lines and Sex and Gender in Research

Cell line source(s)

4662 murine PDAC cell line was derived from a spontaneous pancreatic cancer in a female KPC mouse on the C57BL/6J background (Evans et al., 2016). HEK293T cells were purchased from Clontech #632180.

Authentication

4662 cell lines were tested and authenticated by the Research Animal Diagnostic Laboratory (RADIL) at the University of Missouri using the Infectious Microbe PCR Amplification Test (IMPACT). HEK293T cells were authenticated by the provider.

Mycoplasma contamination

All cell lines tested negative for mycoplasma contamination.

Commonly misidentified lines
(See ICLAC register)

No commonly misidentified lines were used in this study.

Animals and other research organisms

Policy information about studies involving animals; ARRIVE guidelines recommended for reporting animal research, and Sex and Gender in Research

Laboratory animals

C57BL/6, C57BL/6-Tg(TcraTcrb)1100Mjb/J (OT-I), and NOD/SCID mice were purchased from the Jackson Laboratory and/or bred at the University of Pennsylvania. Mice were housed under a 12h-12h light-dark cycle, temperature of 18–23℃, humidity of 36–56%, and pathogen-free conditions.

Wild animals

No wild animals were used in this study.

Reporting on sex

Female mice were used in the study.

Field-collected samples

No field-collected samples were used.

Ethics oversight

All animal procedures used in this study were performed following the National Institutes of Health guidelines. All mouse procedure protocols used in this study were in accordance with, and with the approval of, the Institutional Animal Care and Use Committee (IACUC) of the University of Pennsylvania (protocols 804643 and 805650).

Note that full information on the approval of the study protocol must also be provided in the manuscript.

Flow Cytometry

Plots

Confirm that:

- [x] The axis labels state the marker and fluorochrome used (e.g. CD4-FITC).
- [x] The axis scales are clearly visible. Include numbers along axes only for bottom left plot of group (a 'group' is an analysis of identical markers).
- [x] All plots are contour plots with outliers or pseudocolor plots.
- [x] A numerical value for number of cells or percentage (with statistics) is provided.

Methodology

Sample preparation	Transplant tumors were minced and digested in DMEM supplemented with 2 mg/ml of collagenase type IV (GIBCO, 17104-019) and 0.2 mg/ml of DNase I (Sigma, 10104159001) at 37℃ for 45 minutes and filtered through a 70-μM cell strainer to generate single cell suspensions. Cells were then stained with fluorescence-conjugated antibodies and a live/dead stain (Invitrogen, L34966) at 4℃ for 20 minutes and washed twice with cold PBS plus 5% FBS for sample acquisition. For intracellular caspase staining, cells were further permeabilized with Fix/Perm buffer (eBioscience, 00-5523-00) at 4℃ for 30 minutes and stained with active caspase-3 Ab (C92-605; BD Biosciences) in Perm buffer (eBioscience) at 4℃ for 30 minutes.
Instrument	LSR II (BD Biosciences) was used for flow data acquisition. FACSAria II (BD Biosciences) was used for cell sorting.
Software	FACSDiva v8.0 (BD Biosciences) and FlowJo v10.8.1 (Tree Star) were used for data acquisition and analysis.
Cell population abundance	The purity of post-sort OT-I cells was at a range of 96-99%.
Gating strategy	Singlets were gated based on FSC-A and FSC-H. FSC-A, SSC-A, and Live/dead staining were used to exclude debris and dead cells. Boundaries between positive and negative staining cell populations were determined using isotype control antibodies or FMO.

☒ Tick this box to confirm that a figure exemplifying the gating strategy is provided in the Supplementary Information.
